# Chitin Scaffolds in Tissue Engineering

**DOI:** 10.3390/ijms12031876

**Published:** 2011-03-15

**Authors:** Rangasamy Jayakumar, Krishna Prasad Chennazhi, Sowmya Srinivasan, Shantikumar V. Nair, Tetsuya Furuike, Hiroshi Tamura

**Affiliations:** 1 Amrita Center for Nanosciences and Molecular Medicine, Amrita Institute of Medical Sciences and Research Centre, Cochin-682041, India; E-Mails: drkrishnaprasadc@aims.amrita.edu (K.P.C.); sowmyasrinivasan@aims.amrita.edu (S.S.); shantinair@aims.amrita.edu (S.V.N.); 2 Faculty of Chemistry, Materials and Bioengineering, Kansai University, Osaka-564-8680, Japan; E-Mail: furuike@kansai-u.ac.jp (T.F.)

**Keywords:** chitin scaffold, bone, cartilage, wound, tissue engineering, biomaterials, hydrogel

## Abstract

Tissue engineering/regeneration is based on the hypothesis that healthy stem/progenitor cells either recruited or delivered to an injured site, can eventually regenerate lost or damaged tissue. Most of the researchers working in tissue engineering and regenerative technology attempt to create tissue replacements by culturing cells onto synthetic porous three-dimensional polymeric scaffolds, which is currently regarded as an ideal approach to enhance functional tissue regeneration by creating and maintaining channels that facilitate progenitor cell migration, proliferation and differentiation. The requirements that must be satisfied by such scaffolds include providing a space with the proper size, shape and porosity for tissue development and permitting cells from the surrounding tissue to migrate into the matrix. Recently, chitin scaffolds have been widely used in tissue engineering due to their non-toxic, biodegradable and biocompatible nature. The advantage of chitin as a tissue engineering biomaterial lies in that it can be easily processed into gel and scaffold forms for a variety of biomedical applications. Moreover, chitin has been shown to enhance some biological activities such as immunological, antibacterial, drug delivery and have been shown to promote better healing at a faster rate and exhibit greater compatibility with humans. This review provides an overview of the current status of tissue engineering/regenerative medicine research using chitin scaffolds for bone, cartilage and wound healing applications. We also outline the key challenges in this field and the most likely directions for future development and we hope that this review will be helpful to the researchers working in the field of tissue engineering and regenerative medicine.

## 1. Introduction

Chitin, extracted primarily from shellfish sources, is a unique biopolymer based on the *N*-acetyl-glucosamine monomer. It is a co-polymer of *N*-acetyl-glucosamine and *N*-glucosamine units randomly or block distributed throughout the biopolymer chain depending on the processing method used to derive the biopolymer. When the number of *N*-acetyl-glucosamine units is higher than 50%, the biopolymer is termed chitin. Chitin, together with its derivatives, has been shown to be useful as a wound dressing material, drug delivery vehicle and an essential candidate for tissue engineering. The promise for this biomaterial is vast and will grow as and when the chemistry extends its capabilities with further investigations of new biomedical applications. The present generation of tissue engineering/regenerative medicine research seeks to reinstate the function of injured tissues through the seeding of cells onto porous biodegradable polymer matrixes. In most of the cases, including the bone and cartilage systems, function relies on transmission or generation of mechanical forces and maintenance of blood circulation. Tissues such as articular cartilage have specialized structure and composition which help to provide mechanical and transport properties. As of now, there are only a few engineered tissue products available for clinical use. Most of the implants fail because of the transport limitations leading to death of the implanted cells. The extent of mechanical stresses that tissues may be subjected to, under *in vivo* conditions can be quite large and there are hardly any engineered tissue constructs which possess the properties to withstand such trauma at the time of implantation. Moreover, the challenge is not limited to match a single mechanical parameter, such as elastic modulus or strength; rather, most tissues have intricate viscoelastic, nonlinear and anisotropic mechanical properties that may vary with age, site of implantation and a multitude of other factors. A primary limiting factor in tissue engineering research is the availability of suitable biomaterials to serve as the temporary matrix. These biomaterials must be capable of being prepared in various shapes and sizes with sufficient porosity to offer a channel for the migration of host cells into the matrix, thus permitting growth into complete tissue analogs and be biodegradable generating non-toxic products once they have served their function *in vivo*.

Chitin is a biodegradable polymer and it can be easily processed into membranes [1–8], nanofibers [9,10], gels [11–13] and scaffold [14–16] forms. Chitin scaffolds and membranes have been reported to be suitable for bone [17–21] and wound [22–25] tissue engineering applications. This review presents an overview of the more recent research area of orthopedic, bone and wound tissue engineering applications of chitin scaffolds.

## 2. Chitin Scaffolds in Tissue Engineering

Tissue engineering is a multidisciplinary science, encompassing diverse fields like materials engineering and molecular biology with efforts to develop biological substitutes for failing tissues and organs. Tissue engineering thus seeks to replace diseased and damaged tissues of the body. For successful tissue regeneration, it is absolutely indispensable not only to have cells of high proliferation and differentiation potential, but also to create an environment suitable for inducing regeneration. Such creation can be artificially achieved only by providing various biomaterials to promote cell proliferation and differentiation along with proper cells and growth factors. A number of biodegradable polymers have been explored for tissue engineering purposes. These include synthetic polymers like poly(caprolactone), poly(lactic-co-glycolic acid), poly(ethylene glycol), poly(vinyl alcohol) and natural polymers like alginate, collagen, gelatin, chitin and chitosan *etc.* [26,27]. Of these, chitin and its derivatives have shown tremendous promise as tissue supporting materials.

### 2.1. Bone

Growth of functional tissues include regeneration of specific cells in tissues by facilitating the differentiation of stem cells, or the attachment and proliferation of particular cell types in judiciously designed nanomaterial-based assemblies. Regeneration is based on the hypothesis that healthy progenitor cells either recruited or delivered to an injured site, can eventually regenerate lost or damaged bone tissue. Tissue engineering approaches attempt to create tissue replacements by culturing cells onto synthetic three-dimensional polymer matrixes [28]. Three-dimensional porous polymeric matrixes are seen as one approach to enhance bone regeneration by creating and maintaining channels that facilitate progenitor cell migration, proliferation, and differentiation [29]. The requirements that must be satisfied by such matrixes include providing a space with the proper size and shape for tissue development and permitting cells from the surrounding tissue to migrate into the matrix [30]. The surface of the polymer would also ideally promote cell attachment, as many cells are anchorage dependent for their survival.

The polymer with a mineral to provide a “composite” material that has the toughness and flexibility of the polymer with the strength and hardness of the mineral filler has its beginnings in nature. Recently, “composites” based on polymer matrix-calcium-based compounds systems are of potential use as hard tissue substitute materials. The advantage of such “composites” was believed to be the enhancement of the osteogenic potential due to the presence of calcium compounds and the binder characteristics of the polymer matrix in inhibiting migration of the calcium compounds. For example, the combination of polymer with hydroxyapatite (HA) has been shown to maximize the osteo-conductive behavior of HA *in vivo*, allowing bony in-growths to occur into the implant as the matrix progressively resorbs [31,32]. Therefore, HA or other calcium containing materials incorporated into chitin have been the primary research area where orthopedic or bone substitution and periodontal applications were the focus. In a series of studies, Wan *et al*. have investigated the interaction of chitin with calcium species. In one strategy, Wan *et al*. combined chitin with HA to produce a new HA-chitin “composite” material by dispersing HA into chitin to give an intimately blended material [33]. Preliminary mechanical test results revealed a reduction in strength for the more highly filled composites, but they also revealed retention of the plastic properties of the polymer that may be favorable for bone replacement applications. Wan *et al*. also investigated the induction of *in situ* calcification by chitin. It was theorized that if chitin was chemically modified with anionic carboxymethyl (CM) groups, a chitin matrix with surface acidic CM residues becoming a calcium ion attractor site, could be prepared [34]. If the chitin matrix is placed in the presence of calcium and phosphate ions, the CM chitin would draw calcium ions to it. The calcium ions in turn would pull phosphate ions, forming the basis of calcium phosphate nucleation that eventually would give rise to calcium apatite on the chitin platform. The results of this preliminary work indicated that higher the CM content, the better the calcification, suggesting a role for *in vivo* calcification promotion by chitin. Subsequently, the nucleation idea was refined by preparing the chitin in the form of porous chitin matrixes obtained by freeze-drying chitin gels followed by chemically modifying their surfaces with CM and phosphoryl (P-chitin) ionic groups [35,36]. The more porous nature of this method of preparation of the chitin matrix was thought to provide more opportunity for “through matrix” calcium ions attraction that would lead to more substantial calcium phosphate-chitin material. The results from these studies indicated that a calcium phosphate phase could be efficiently grown onto a pre-formed chitin template by incubating in a metastable solution supersaturated with respect to calcium phosphate. High levels of calcium phosphate (54.6% of final composite mass) were incorporated into the porous chitin matrix (Ca_P = 8 mm^2^). An average of 19.5% by mass of calcium phosphate (as percentage of final composite mass) was deposited on the CM chitin, under the same conditions. In this study the P-chitin was found to have an inhibitory effect on calcium ion recruitment.

One of the most attempted methods to prepare chitin for cell seeding is to first make a precursor that is typically a gel followed by various lyophilization strategies. A series of porous chitin matrixes were obtained by producing chitin gels from chitin solutions further lyophilized to give porous chitin matrixes [35–37]. Matrix pore sizes ranging from 100 to 500 mm were obtainable depending on the various pre-treatment procedures of chitin gels prior to lyophilization [37]. Mouse and human fibroblast cell cultures exposed to these chitin matrixes were found to grow and proliferate well indicating the feasibility of using these porous chitin matrixes for cell transplantation applications to regenerate tissues. Similarly, Wang *et al*. have demonstrated the preparation of chitin-plasma sprayed calcium HA matrixes [38]. HA in 25, 50 and 75% w/w fractions was incorporated into chitin solutions and air and freeze-dried to develop porous like scaffold [39]. These HA-chitin porous scaffolds were exposed to cell cultures and implanted into the intramusculature of a rat model. The HA-chitin scaffold were found to be non-cytotoxic and degraded *in vivo*. The presence of the HA filler enhanced calcification as well as accelerated degradation of the chitin matrix. The prepared HA-chitin matrixes were selected for further cell seeding experiments. Mesenchymal stem cells harvested from NZW rabbits differentiated into osteoblasts *in vitro* using dexamethasone. These osteoblasts were cultured for one week, statically loaded onto the porous HA-chitin matrixes and implanted into bone defects of the rabbit femur for two months. Histology of explants showed bone regeneration with biodegradation of the HA-chitin matrix. Similarly, green fluorescence protein (GFP) transfected MSC-induced osteoblasts were also loaded onto porous HA-chitin matrixes and implanted into the rabbit femur. The results from GFP-transfected MSCs showed that loaded MSCs-induced osteoblasts did not only proliferate but also recruited the ingrowth of surrounding tissue. These results indicated that HA-chitin matrixes could be good candidates for tissue engineered bone substitute [39].

A CMC/HA composite was prepared and examined for their effects on bone repair in cell lines and in animals [40]. The efficiency of bone formation by CMC/HA composite was compared with CMC, HA or blank by implantation of those materials in animals *in vivo*. The new bone formation of CMC/HA composite was superior to that of CMC, HA or blank. Thus, a porous CMC/HA composite could act as a scaffold for osteoblast-like cells and a barrier to in growth of fibrous connective tissues. The cytotoxicity of CMC was evaluated using the MC3T 3-E1 cell line. Cells had very low toxicity when the DD of CMC was 0.1%. It is evident that the control of DD is a very important factor in using CMC as a bone repair material. The CMC/HA composite showed high blood absorbing property and was tightly fixed in the bone defect without flowing out during periosteum suturing, while HA was easy to flow out of the bone defect by bleeding from the bone marrow cavity [40]. β-chitin hydrogel scaffolds were also developed by lyophilization technique [14]. The bioactivity studies of β-chitin scaffolds studied using SBF solution showed that there is a calcium phosphate layer on the surface as well as in the cross section of β-chitin scaffolds. It appears that β-chitin scaffolds can also be used for bone tissue engineering [14].

nHA with the chemical formula Ca_10_(PO_4_)_6_(OH)_2_ is commonly used for various biomedical applications owing to its unique functional properties of high surface area to volume ratio and it also mimics the apatite like structure and composition of hard tissues such as enamel, dentin and bone [39,41]. It is stable in body fluids, bioactive, osteoconductive, non-toxic, non inflammatory, non immunogenic and does not decompose [42,43]. It has the ability to form a direct chemical bond with the surrounding hard tissues [44]. In addition it offers other advantages such as easy fabrication, handling and close surface contact with the surrounding tissues [45]. β-chitin/HA nanocomposite scaffolds were synthesized from a mixture of β-chitin hydrogel and nHA by freeze-drying technique [46]. The cytocompatibility of the nanocomposite scaffolds was studied using MG 63 cells. The results indicated that the cells were viable and showed enhanced attachment and proliferation onto the nanocomposite scaffolds. Similar results were observed with α-chitin/HA nanocomposite scaffolds synthesized from α-chitin hydrogel and nHA by freeze-drying approach [47]. These results essentially signify that the synthesized nanocomposite scaffolds can serve as potential candidates for bone tissue engineering [46,47].

Bioactive glass ceramics are silicate-based materials used for bone repair and are widely used in orthopedics and dentistry [48,49]. They can also bond to both soft and hard tissue and are superior to HA in their ability for osseointegration and osteoblast and marrow stromal cell proliferation and differentiation [50–56]. In addition, the bioactive glass ceramics influence osteoblastic cell differentiation with an increase in the level of differentiation markers like ALP, osteocalcin and osteopontin [55,56]. Bioactive glass ceramic nanoparticles (nBGC) were prepared using sol-gel technique [16]. The α-chitin/nBGC and β-chitin/nBGC composite scaffolds were prepared using α-chitin/β-chitin hydrogel with nBGC by lyophilization technique [16,17]. The biocompatibility of the composite scaffolds was studied using MTT assay and cell attachment studies on MG63 and POB cells. Results indicated no signs of toxicity and the MG63 and POB cells were well adhered onto the scaffolds. These results suggested that the developed composite scaffolds have promising applications in alveolar bone tissue engineering [16,17].

Addition of silica nanoparticles enhances the bioactivity and biocompatibility of chitin. Recently, α-chitin composite scaffold containing nanosilica was developed using α-chitin hydrogel [15]. Bioactivity, swelling ability and cytotoxicity of α-chitin composite scaffolds were analyzed *in vitro*. These scaffolds were found to be bioactive in SBF and biocompatible with MG63 cell line. The α-chitin/nanosilica composite scaffolds showed higher biocompatibility. These results suggest that α-chitin/nanosilica composite scaffolds can be useful for bone tissue engineering [15].

Polymeric nanofibrous scaffolds can mimic the structure and function of the natural extracellular matrix (ECM). These materials are of great interest in tissue engineering as scaffolding materials to restore, maintain or improve the function of human tissues. The natural ECMs in the body are mainly composed of two classes of extracellular macromolecules: proteoglycans and fibrous proteins with fiber diameter ranging from 50 to 150 nm, depending on the tissue type [57]. Recent studies showed that the material size feature could substantially influence the morphology and function of cells grown on the ECM. The attachment and proliferation of cells were found to be good on micro and nanostructured materials [58,59]. Collagen is a major natural extracellular matrix component and possesses a fibrous structure with fiber bundles varying in diameter from 50 to 500 nm [58,60]. Much effort has been made to find an alternative scaffold material with similar physicochemical and biological characteristics of ECM [61]. The morphology of electrospun nanofiber mat is very similar to the morphology of human native ECM [62–64] thus; electrospun nanofiber could be a promising scaffolding material for cell culture and tissue engineering application. The electrospinning process makes it possible to produce complex, seamless and three-dimensional (3D) nanofibrous scaffolds that support diverse types of cells to form artificial tissues.

Electrospun water-soluble carboxymethyl chitin (CMC)/PVA blend nanofibrous scaffold for tissue engineering applications has been reported [10]. The prepared nanofibers were found to be bioactive and biocompatible. Cytotoxicity and cell attachment studies of the nanofibrous scaffold were evaluated using human mesenchymal stem cells (hMSCs) by the MTT assay. Cell attachment studies revealed that cells were able to attach and spread throughout the nanofibrous scaffolds. These results indicated that the nanofibrous CMC/PVA scaffold support cell adhesion/attachment and proliferation and hence this scaffold can be a useful candidate for bone tissue engineering [10].

### 2.2. Cartilage

Articular cartilages have distinctive mechanical properties to withstand the demands of repetitive loading. However, this tissue exhibits little or no wear, even with recurring loading cycles that may even exceed 8–12 times the body weight of the individual. This inimitable property of the cartilage tissue has been attributed to its complex structure and unique extra cellular matrix composition, which provides mechanical properties that are anisotropic, nonlinear, inhomogeneous and viscoelastic [65]. Additionally, the fixed negative charge maintained in the extra cellular matrix due to the high concentration of aggrecan and other proteoglycans increases the internal swelling pressures providing inhomogeneous residual stresses within normal articular cartilage.

Chitin has the ability to maintain the morphology of chondrocytes, a normal phenotypic characteristic and preserve their capacity to synthesize cell-specific extracellular matrix. Scaffolds made from pure β-chitin showed the efficiency in supporting chondrocytes (*ca.* 98%) and the same concentration of chondroitin sulfate [66]. The content of hydroxyproline in the β-chitin sponge scaffold was significantly greater than in other sponges at week 4, post-culture. From the histochemical and immuno-histochemical findings, the cartilage-like layer in the chondrocytes-sponge composites was similar to hyaline cartilage. However, only in the pure β-chitin sponge scaffold, type II collagen was closer to that of normal rabbit cartilage [66]. These results indicated that pure β-chitin sponge scaffold can be used for cartilage tissue engineering.

### 2.3. Wound

Chitin has an accelerating effect on the wound healing process. The main biochemical activities of chitin based materials in wound healing are polymorphonuclear cell activation, fibroblast activation, cytokine production, giant cell migration and simulation of type IV collagen synthesis [67]. The cytocompatibility of chitin nanofibrous scaffold was studied for wound tissue engineering applications [68]. Chitin nanofibous scaffolds were found to promote cell attachment and spreading of normal human keratinocytes and fibroblasts compared to chitin microfibers. This may be a consequence of the high surface area available for cell attachment due to their three-dimensional features and high surface area to volume ratios, which are favorable parameters for cell attachment, growth and proliferation. Cell studies conducted on chitin/PGA [69] and chitin/SF [70] fibrous scaffolds proved that a matrix consisting of 25% PGA or SF and 75% chitin had the best results. The chitin/PGA fibrous scaffold had a bovine serum albumin coating and was considered as a good candidate for use as a tissue engineering scaffold. The chitin/SF fibrous scaffold had the highest spreading of normal human epidermal fibroblasts (NHEF) and normal human epidermal keratinocytes (NHEK). Therefore, these scaffolds were recommended for wound tissue engineering applications.

β-chitin/HA nanocomposite scaffolds were synthesized from a mixture of β-chitin hydrogel and nHA by freeze-drying technique [46]. The cytocompatibility of the nanocomposite scaffolds was studied using Vero, NIH 3T3 and HDF cells. The results indicated non-toxicity with enhanced attachment and proliferation of these cells onto the nanocomposite scaffolds. Similar results were observed with α-chitin/HA nanocomposite scaffolds synthesized from α-chitin hydrogel and nHA by freeze-drying approach [47]. These results essentially signify that the synthesized nanocomposite scaffolds can serve as potential candidates for wound tissue engineering [46,47].

Recently, β-chitin/nanosilver composite scaffolds were prepared for wound tissue engineering applications using β-chitin hydrogel with silver nanoparticles [24]. The antibacterial, blood clotting, swelling, cell attachment and cytotoxicity studies of the prepared composite scaffolds were evaluated. The prepared β-chitin/nanosilver composite scaffolds were bactericidal against *E. coli* and *S. aureus* and showed good blood clotting ability as well. Cell attachment studies using Vero (epithelial cells) showed that the cells were well attached on the scaffolds. Similarly, α-chitin/nanosilver composite scaffolds showing similar results were developed for wound tissue engineering applications using α-chitin hydrogel with silver nanoparticles [25]. These results suggested that chitin/nanosilver composite scaffolds could be potential candidates for wound tissue engineering [24,25].

## 3. Conclusions

There has been a remarkable progression during the past two decades in the development of tissue engineering scaffolds which can be attributed in large measure to novel advanced materials. For regeneration of complex human organs, highly porous and fortified 3D molds are indispensable. Recently, hydrogel scaffold such as chitin is greatly used for improving the functionality of tissue-engineered constructs. Chitin has excellent properties such as biodegradability, bio-compatibility, non-toxicity and have been shown to increase wound healing in animals and humans. Chitin has also demonstrated a physiological compatibility with living tissues. In addition, basic and applied research has made great progress and it is evident that chitin exhibits an unlimited application potential for use in bone, cartilage and wound care. Probably, the only limitation of chitin is the poor mechanical strength and elasticity to serve as scaffold for tissues like bone and cartilage. However, the mechanical property can be improved with the addition of biomaterials like hydroxyapatite (HA), bioactive glass ceramic (BGC), *etc*. and thus can be explicitly designed and tuned to match native tissues.

The examples described in this review represent only a few of the numerous ways in which chitin can be used to design scaffolds for bone and cartilage tissue engineering. Similar concepts could be thought of for regenerating other tissues or organs using chitin scaffolds. Also, the uniqueness of the chitin both as 3D scaffolds and as an injectable hydrogel functionalized with desired biomolecules can be widely explored for various tissue engineering applications. Further studies are needed to demonstrate the potential uses of chitin scaffold in the tissue engineering/regenerative medicine area. In the area of tissue engineering and regenerative medicine, there continues to be a huge pressure to develop advanced materials that can better mimic the exquisite architecture and functional properties of native tissues. In the near future, it is most likely that the hydrogel based systems would help to reconcile the clinical and commercial demands in tissue engineering.
